# Treatment of asymptomatic carriers with artemether-lumefantrine: an opportunity to reduce the burden of malaria?

**DOI:** 10.1186/1475-2875-9-30

**Published:** 2010-01-22

**Authors:** Bernhards Ogutu, Alfred B Tiono, Michael Makanga, Zulfiqarali Premji, Adama Dodji Gbadoé, David Ubben, Anne Claire Marrast, Oumar Gaye

**Affiliations:** 1 Walter Reed Project/Centre for Clinical Research-Kenya Medical Research Institute, Nairobi, Kenya; 2 Malaria Clinical Trials Alliance - INDEPTH Network, Nairobi, Kenya; 3 Centre National de Recherche et de Formation sur le Paludisme (CNRFP), Ouagadougou, Burkina Faso; 4 European and Developing Countries Clinical Trials, Cape Town, South Africa; 5 Department of Parasitology and Medical Entomology, Muhimbili University College of Health Sciences, Dar es Salaam, Tanzania; 6 Department of Paediatrics, University of Lomé, Lomé, Togo; 7 Medicines for Malaria Venture, Geneva, Switzerland; 8 Novartis Pharma AG, Basel, Switzerland; 9 Faculty of Medicine, University CAD Dakar, Senegal

## Abstract

**Background:**

Increased investment and commitment to malaria prevention and treatment strategies across Africa has produced impressive reductions in the incidence of this disease. Nevertheless, it is clear that further interventions will be necessary to meet the international target of a reversal in the incidence of malaria by 2015. This article discusses the prospective role of an innovative malaria control strategy - the community-based treatment of asymptomatic carriers of *Plasmodium falciparum*, with artemisinin-based combination therapy (ACT). The potential of this intervention was considered by key scientists in the field at an Advisory Board meeting held in Basel, in April 2009. This article summarizes the discussions that took place among the participants.

**Presentation of the hypothesis:**

Asymptomatic carriers do not seek treatment for their infection and, therefore, constitute a reservoir of parasites and thus a real public-health risk. The systematic identification and treatment of individuals with asymptomatic *P. falciparum *as part of a surveillance intervention strategy should reduce the parasite reservoir, and if this pool is greatly reduced, it will impact disease transmission.

**Testing the hypothesis:**

This article considers the populations that could benefit from such a strategy and examines the ethical issues associated with the treatment of apparently healthy individuals, who represent a neglected public health risk. The potential for the treatment of asymptomatic carriers to impair the development of protective immunity, resulting in a 'rebound' and age escalation of malaria incidence, is also discussed.

For policymakers to consider the treatment of asymptomatic carriers with ACT as a new tool in their malaria control programmes, it will be important to demonstrate that such a strategy can produce significant benefits, without having a negative impact on the efficacy of ACT and the health of the target population.

**Implications of the hypothesis:**

The treatment of asymptomatic carriers with ACT is an innovative and essential tool for breaking the cycle of infection in some transmission settings. Safe and effective medicines can save the lives of children, but the reprieve is only temporary so long as the mosquitoes can become re-infected from the asymptomatic carriers. With improvements in rapid diagnostic tests that allow easier identification of asymptomatic carriers, the elimination of the pool of parasites is within reach.

## Background

The last two decades have seen an increasing level of international attention directed towards malaria. In 2000, the Millennium Development Goals called for a reversal in the incidence of this disease by 2015. Following the publication of this ambitious goal, an increased commitment to a number of treatment and prevention strategies has produced some impressive results. In Rwanda, Ethiopia, and Zanzibar, the mass distribution of insecticide-treated bed nets (ITNs), coupled with increasing use of long-lasting ITNs and nationwide adoption of artemisinin-based combination therapy (ACT), resulted in substantial declines in malaria-related deaths [1,2]. Similarly, interventions to increase the coverage of ITNs in The Gambia and to improve vector control through a programme of indoor residual spraying (IRS) in South Africa have both produced marked reductions in confirmed malaria cases [3,4]. Despite these considerable successes, it is clear that additional complementary interventions are still required to further reduce the disease burden in areas that have already achieved low transmission. In this situation, the use of surveillance for systematic treatment of asymptomatic cases becomes an intervention of choice.

In the current climate of considerable international attention and funding for the control of malaria, it is important to strive for the implementation of integrated, novel interventions. If substantial headway is not made in controlling malaria in the coming years, it is possible that the attention of donors and funding agencies may drift to other diseases. It is, therefore, critical to seize the current opportunity to implement pioneering, co-ordinated strategies that have a high likelihood of producing an impact. This article proposes that the treatment of asymptomatic carriers represents an opportunity to reduce the burden of malaria.

### The prevalence of asymptomatic carriage of *Plasmodium falciparum*

In malaria-endemic countries, a large proportion of *P. falciparum *infections are asymptomatic or sub-clinical. Microscopy-detected levels of asymptomatic carriage as high as 39% have been reported (Table 1) [5-9]. Invariably, this hidden pool of parasites is essential for maintaining the cycle of infection. Asymptomatic carriers do not seek treatment for their infection and, therefore, constitute a reservoir of parasites available for transmission by mosquitoes. Even in areas of highly seasonal malaria transmission, where the incidence of clinical malaria is significantly lower during the dry season, studies have shown that a considerable proportion of the population remain positive for parasitaemia throughout the year [6,8,10,11]. Indeed, these reports might underestimate the level of asymptomatic carriage during the dry season, as gametocytes have been detected in prolonged sub-microscopic infections [12,13]. Interestingly, the prevalence of asymptomatic carriage has been reported to be greatest in adolescents and children; in a study in The Gambia, the prevalence of asexual parasitaemia (61%) was highest in individuals aged 5-15 years [8].

**Table 1:** Prevalence of asymptomatic carriage of *P. falciparum *in sub-Saharan Africa

	**Gabon **[7]	**Kenya **[9]	**Mozambique **[5]	**Senegal **[6]	**The Gambia **[8]
**Population studied**	Adult males 18-51 years old	Children 5-17 years old	Children under 10 years old	Children 2-10 years old	Individuals >6 months old
**Prevalence of asymptomatic carriage (method of detection)**	12% (microscopy) 27% (RDT) 52% (PCR)	12.6% (microscopy) 33.3% (RDT)	39.2% (microscopy)	35% (microscopy)	32% (microscopy)

## Presentation of the hypothesis

### Reducing the parasite reservoir

On the basis of these measurements, it is clear that the systematic identification and treatment of asymptomatic carriers, as part of a surveillance strategy towards malaria elimination, could reduce the pool of parasites available for the infection of mosquitoes. Therefore, if a significant reduction of the malaria parasite pool could be obtained through treatment of asymptomatic carriers, over a period of time, a reduction in disease transmission could be obtained across the entire endemic population, even in areas of high transmission.

The transmission of *P. falciparum *parasites from humans to mosquitoes requires the presence of infectious gametocytes in the human peripheral blood. Consequently, for the treatment of asymptomatic carriers to result in a reduction of disease transmission, the therapy used must have some effectiveness against the transmissible sexual stage of the parasite. In comparison with non-artemisinin regimens, treatment with artemisinin derivatives has been shown to result in lower gametocyte carriage rates [14,15], and reduced infectivity of treated individuals [16].

Among the ACT currently on the market, artemether-lumefantrine is a good candidate for the treatment of asymptomatic carriers for a number of important reasons. The efficacy and safety of this ACT have been clearly demonstrated in a clinical development programme spanning 14 years and enrolling approximately 5,000 patients. In trials conducted in different malaria-endemic regions, artemether-lumefantrine has consistently achieved 28-day PCR-corrected cure rates of >95% [17-21], and has shown a good safety and tolerability profile in infants, children and adults [17-22].

### Treatment of confirmed carriers not preventive treatment

It is important that any discussion of the treatment of asymptomatic carriers clearly differentiates this strategy from mass drug administration (MDA), as employed for filariasis, or intermittent preventive treatment (IPT) of malaria. MDA involves blanket treatment of the entire endemic population. During IPT, members of an at-risk population are given a full therapeutic dose of treatment at set times, without an attempt to determine infection status. By contrast, a strategy of treating asymptomatic carriers would involve confirmation of parasitaemia using Rapid Diagnostic Tests (RDTs) before treatment. The current deployment of RDTs across sub-Saharan Africa provides a feasible platform for the diagnosis and treatment of the majority of asymptomatic carriers. In stark contrast to policies of MDA, which caused fears over resistance development[23], only individuals with confirmed parasitaemia would receive treatment and this would be a full therapeutic course of treatment, minimizing unnecessary drug use and limiting the risk of any parasite resistance developing to the ACT.

RDT false negativity due to very low parasitaemia or parasite genetic polymorphisms is a possible limitation of RDT screening, as is the difficulty of reaching all members of a community. The most sensitive method for detecting parasitaemia is PCR; however, this technique requires specific equipment that is largely unavailable in rural communities. Identification of asymptomatic carriers through a programme of RDT testing and re-testing is a pragmatic approach, which should reduce the parasite reservoir to a level sufficient to interrupt disease transmission. With repeated surveys and active asymptomatic case detection at health facilities, sufficient levels of treatment could be obtained. As more robust and user-friendly diagnostics become available they could be integrated into the surveillance strategy.

### Preserving the efficacy of ACT

One of the challenges likely to be raised to the treatment of asymptomatic carriers is the potential for such a strategy to engender the development of resistance to ACT. A recent study indicates that the probability of resistance selection in asymptomatic infections is lower than with symptomatic infections. The requirement for a resistant parasite to derive from a new antigenic variant, and the much lower total number of parasites present in an asymptomatic infection lower the probability of a *de-novo *resistant parasite emerging and reaching a transmissible density [24].

Furthermore, the relatively short half-life of lumefantrine makes artemether-lumefantrine an ideal choice for use in this strategy. At approximately 3-6 days, the half-life of lumefantrine is markedly shorter than those of other artemisinin companion drugs, such as mefloquine (2-3 weeks), chloroquine (8-58 days) and piperaquine (2-3 weeks) [25-27]. Resistance to anti-malarial drugs is most likely to occur when a large population of parasites are exposed to sub-therapeutic concentrations of a single anti-malarial. Rapidly eliminated drugs, such as the artemisinin derivatives, cannot protect the partner drug once blood concentrations have fallen below therapeutic levels. Consequently, those ACT that contain partner drugs with a long half-life should, theoretically, be at a greater risk for the development of resistance. Notably, a study that examined the pharmacokinetic determinants of resistance selection showed lumefantrine to have a considerably shorter 'window of selection' than mefloquine and chloroquine [28].

The use of a surveillance strategy for the treatment of asymptomatic carriers, once a state of low malaria transmission is achieved, will ultimately result in a reduction in the disease burden. The proportion of the population exposed to anti-malarials over time will, therefore, be reduced, limiting the possibility of a drug pressure effect that could drive the emergence of resistance to anti-malarials.

Modelling of parasite dynamics with respect to drug resistance would be an important part of any pilot implementation of this strategy, and the effects of other malaria control interventions, both current and future, would need to be considered appropriately.

## Testing the hypothesis

### Defining the population

In sub-Saharan Africa, many of the preventive interventions, such as provision of ITNs and IPT, have focused on those populations most vulnerable to malaria mortality: children under five years of age and pregnant women [29]. For these particular interventions, an approach that targets the high-risk groups is plausible and appropriate, given the limited resources available and with increased survival rather than disease elimination as the main outcome. The ultimate aim of surveillance and treatment of asymptomatic carriers, however, is eradication of disease transmission, which requires screening of all members of a population. The primary intention of the strategy is to dramatically reduce the pool of parasites available for infection of mosquitoes. To break the cycle of transmission, high parasite clearance rates would need to be attained across all members of a community, not only the vulnerable populations.

Although the treatment of asymptomatic carriers would have benefits in all transmission settings, it is likely that the initial proof of concept would be easiest to achieve in areas of highly seasonal transmission. In these areas, the continuing cycle of infection is very heavily dependent on asymptomatic carriage of parasites during the dry season. Consequently, treatment of asymptomatic carriers before the start of the rainy season and dry season could dramatically reduce the source of *P. falciparum *infection for newly emerging mosquitoes. In high malaria transmission areas such as Rwanda, South Africa and Zanzibar, which have already achieved a marked reduction in the disease burden following scale-up of ITNs, IRS, ACT and IPT, this could be an appropriate strategy to further reduce the disease burden and move towards parasite elimination.

### Measuring the benefits

As adolescents and adults experience far fewer episodes of clinical malaria, the primary benefits of this strategy would need to be measured in the most vulnerable members of the population: children less than five years of age. With a reduction in disease transmission, children under five years of age would experience fewer cases of clinical malaria with improved survival and quality of life.

To convince policymakers of the potential benefits of this strategy, any pilot study would need to examine endpoints that are likely to be of interest to this audience. Consequently, while the immediate result of treating asymptomatic carriers will be to reduce the prevalence of asymptomatic carriage, the impact of the strategy on the incidence of clinical malaria in children aged less than five years will be the far more compelling measurement for policymakers.

Cost and affordability will be key factors for policymakers. In this regard, it is likely that the cost of testing individuals and treating asymptomatic carriers would be more than offset by the reduced number of malaria-related hospital admissions and patient visits, and by the man hours gained. In 2002, Muheki *et al *calculated that the average cost of each malaria-related hospital admission in the KwaZulu Natal province of South Africa was (US$) 173.5 and an outpatient visit was $7.52 [30]. With a drop in malaria incidence, these significant healthcare costs could be reduced. However, there are costs that would need to be taken into consideration such as those associated with community education to encourage participation and adherence to treatment, and enhancement of healthcare systems to facilitate implementation of the strategy. All in all, the anticipated gains should outweigh the costs, with the strategy resulting in a reduction in disease burden and improved healthcare systems that have an emphasis on disease prevention. Recent work has demonstrated the pervasive effect of malaria on a country's socioeconomic situation [31]. Consequently, a credible case could be made for evaluating the impact of malaria control interventions from a macro-socioeconomic standpoint. Clearly a full health-economic analysis would be an essential part of the implementation of any pilot study that examines the potential of this intervention.

### The challenges associated with the treatment of asymptomatic carriers

One of the challenges this control strategy would need to address is the need to treat 'apparently' healthy individuals. Indeed, convincing asymptomatic individuals to comply with treatment could prove difficult, as the usual pay-off of symptom resolution with treatment would not be present. It is important to emphasize, however, that while asymptomatic carriers do not present with the symptoms of clinical malaria, they still carry a burden from the disease. Anaemia related to malaria has been recorded in asymptomatic individuals in a number of studies [32-34], and malaria preventive measures have been shown to reduce the prevalence of anaemia in school-aged children [34,35]. Anaemia has been related to reduced work capacity [36], poor pregnancy outcomes [37], and reduced cognitive function [38]. It is, therefore, anticipated that individual carriers would derive benefit from clearance of their parasitaemia.

With the use of appropriate healthcare educational tools to convey the population and individual benefits, and in the knowledge that the uptake of previous mass vaccination programmes by healthy individuals has proven successful - such as the campaign to educate the population regarding the elimination of polio, sufficient uptake of treatment by asymptomatic individuals should be possible. Community involvement in the programme would be vital to encourage high levels of participation. Notably, excellent adherence to the artemether-lumefantrine treatment regimen during clinical malaria has been reported [39,40].

A further potential barrier would be convincing public-health officials of the favourable risk-to-benefit profile of the strategy. In contrast to the treatment of patients with clinical malaria, the benefits conferred by the treatment of asymptomatic carriers would largely be at the population rather than individual level. Therefore, policymakers would need to be convinced that the population-level benefits are sufficiently large to justify the treatment of apparently healthy individuals with anti-malarials. In this regard, it would be extremely important to use an ACT, such as artemether-lumefantrine, for which a good safety and tolerability profile has been clearly established.

Another concern that would need to be addressed is the potential for such a strategy to impair the development of protective immunity. It is known that children living in malaria-endemic areas gradually acquire immunity to the disease, and their risk for clinical malaria is markedly reduced by adolescence. If the development of protective immunity was compromized by the strategy, this might result in a 'rebound' of malaria incidence. On consideration, however, it seems unlikely that the strategy would result in a significant rebound effect. Firstly, the strategy does not prevent the exposure of individuals to parasites; indeed, individuals only receive treatment once it has been confirmed that they have parasitaemia. Secondly, similar concerns were raised with regard to the widespread implementation of IPT strategies and use of ITNs for infants and small children, but subsequent studies have indicated that the rebound effect was not as significant as hypothesized [41-48]. Studies of ITN-type strategies in Burkina Faso, Ghana and Kenya found no evidence to suggest a shift in mortality from younger to older children [41-43]. Similarly, several studies looking for evidence of a rebound effect following IPT have failed to find an increase in the incidence of malaria in the subsequent year [44-48].

While this strategy may alter the profile of malaria immunity, its use is proposed in the context of malaria eradication. In such a setting, the fear of waning malaria immunity and subsequent disease rebound must be balanced against the huge quality of life benefits and opportunities that would be obtained with eradication of the disease. Many individuals in malaria-endemic regions do not reach their full potential, owing to the lifelong consequences of early malaria infections. Studies have shown that IPT strategies, that reduced parasitaemia in children, resulted in better school performance and quality of life [34,49]. These findings cast doubt on the value of retaining parasites in order to maintain immunity at the expense of individuals attaining their full life potential. A significant reduction in disease prevalence should also lead to considerable socioeconomic as well as health-related benefits including improved healthcare systems.

The fundamental aim of the malaria eradication agenda is to eliminate the parasite, which will ultimately result in a loss of immunity for the entire population, since malaria infection does not confer lifelong immunity. For individuals moving to areas still endemic for disease, other treatment and preventive strategies such as prophylaxis would still be available.

Fears over the adverse effects that could result from failure of a malaria elimination programme should not result in the surrender of ambition. The knowledge of such possibilities allows active management of these challenges, and should not prevent the development and deployment of new strategies that are likely to accelerate achievement of the ultimate aim - malaria elimination. Although the programme for malaria eradication failed in Africa previously, it was successful in other areas, and thus with lessons learnt, greater global commitment, and the availability of a greater number of tools, the community must strive for success.

## Implications of the hypothesis

In light of the recent successes associated with the introduction of ITNs, IRS and ACT, and the increased international attention being given to malaria control and prevention it is time to explore new disease-control strategies that can build on the achievements of the past 20 years. The treatment of asymptomatic carriers as part of a surveillance strategy has the potential to make a very significant contribution to the multifaceted approaches being implemented by malaria control programmes across Africa. Accordingly, suitable pilot studies designed to obtain evidence of the benefits of this strategy should now be a priority. These pilot studies are likely to be large, expensive and complex in their design, but are essential to develop this extremely promising concept into a scientifically well-formulated strategy.

As the malaria community begins to seriously consider malaria elimination in certain epidemiological settings, there is a clear need to broaden the research agenda and develop new control interventions [50,51]. The treatment of asymptomatic carriers is one such intervention that would work in conjunction with existing approaches such as prompt and effective case management, IPT and vector control, as well as with future strategies such as a vaccine against malaria.

In conclusion, a co-ordinated surveillance strategy to identify and treat both symptomatic and asymptomatic carriers of *P. falciparum *has great potential to reduce disease transmission towards elimination. Although such a strategy would present a number of operational challenges, if compelling evidence of the benefits of this intervention could be produced, solutions to the implementation barriers would be found, as demonstrated by previous vaccination campaigns.

Effective anti-malarials save lives; however, without a significant reduction in disease transmission, these individuals quickly become reinfected and the cycle continues with the potential for the development of resistance and the loss of lives. With improvements in diagnostic tools, such as RDTs, which allow for easier and more accurate identification of asymptomatic carriers, it is possible that the elimination of the pool of parasites is within reach.

## Abbreviations

ACT: artemisinin-based combination therapy; IPT: intermittent preventive treatment; IRS: indoor residual spraying; ITN: insecticide-treated bed nets; MDA: mass drug administration; PCR: polymerase chain reaction; RDT: rapid diagnostic test.

## Competing interests

BO: I declare that I received a consultancy fee for attendance at an Advisory Board Meeting co-sponsored by Novartis Pharma AG and Medicines for Malaria Venture.

ABT: I declare that I received a consultancy fee for attendance at an Advisory Board Meeting co-sponsored by Novartis Pharma AG and Medicines for Malaria Venture.

MM: I declare that I received a consultancy fee for attendance at an Advisory Board Meeting co-sponsored by Novartis Pharma AG and Medicines for Malaria Venture.

ZP: I declare that I received a consultancy fee for attendance at an Advisory Board Meeting co-sponsored by Novartis Pharma AG and Medicines for Malaria Venture.

ADG: I declare that I received a consultancy fee for attendance at an Advisory Board Meeting co-sponsored by Novartis Pharma AG and Medicines for Malaria Venture.

DU: I am an employee of Medicines for Malaria Venture.

ACM: I am an employee of Novartis Pharma AG, the manufacturer of artemether-lumefantrine (Coartem^®^).

OG: I declare that I received a consultancy fee for attendance at an Advisory Board Meeting co-sponsored by Novartis Pharma AG and Medicines for Malaria Venture.

## Authors' contributions

All authors were involved in the conception of the manuscript and in defining the content. In addition, all authors revised the manuscript critically for important intellectual content, and read and approved the final manuscript.
